# Characterization of complex flow patterns in the ascending aorta in patients with aortic regurgitation using conventional phase-contrast velocity MRI

**DOI:** 10.1007/s10554-017-1239-3

**Published:** 2017-09-04

**Authors:** Odd Bech-Hanssen, Frida Svensson, Christian L. Polte, Åse A. Johnsson, Sinsia A. Gao, Kerstin M. Lagerstrand

**Affiliations:** 1 Departments of Clinical Physiology, The Sahlgrenska Academy at the University of Gothenburg, Gothenburg, Sweden; 2 Departments of Cardiology, The Sahlgrenska Academy at the University of Gothenburg, Gothenburg, Sweden; 3 Departments of Diagnostic Radiation Physics, The Sahlgrenska Academy at the University of Gothenburg, Gothenburg, Sweden; 4 Departments of Radiology, The Sahlgrenska Academy at the University of Gothenburg, Gothenburg, Sweden; 5 Institute of Medicine at the Sahlgrenska Academy, Sahlgrenska University Hospital, Gothenburg, Sweden; 6 Departments of Clinical Sciences, The Sahlgrenska Academy at the University of Gothenburg, Gothenburg, Sweden

**Keywords:** Aortic regurgitation, Cardiovascular magnetic resonance, Flow displacement, Aortic aneurysm

## Abstract

Ascending aorta (AA) flow displacement (FD) is a surrogate for increased wall shear stress. We prospectively studied the flow profile in the AA in patients with aortic regurgitation (AR), to identify predictors of FD and investigate whether magnetic resonance imaging (MRI) phase-contrast flow rate curves (PC-FRC) contain quantitative information related to FD. Forty patients with chronic moderate (n = 14) or severe (n = 26) AR (21 (53%) with bicuspid aortic valve) and 22 controls were investigated. FD was determined from phase-contrast velocity profiles and defined as the distance between the center of the lumen and the “center of velocity” of the peak systolic forward flow or the peak diastolic negative flow, normalized to the lumen radius. Forward and backward volume flow was determined separately for systole and diastole. Seventy percent had systolic backward flow and 45% had diastolic forward flow in large areas of the vessel. AA dimension was an independent predictor of systolic FD while AA dimension and regurgitant volume were independent predictors of diastolic FD. Valve phenotype was not an independent predictor of systolic or diastolic FD. The linear relationships between systolic backward flow and systolic FD and diastolic forward flow and diastolic FD were strong (R = 0.77 and R = 0.76 respectively). Systolic backward flow and diastolic forward flow identified marked systolic and diastolic FD (≥0.35) with a positive likelihood ratio of 6.0 and 10.8, respectively. In conclusion, conventional PC-FRC data can detect and quantify FD in patients with AR suggesting the curves as a research and screening tool in larger patient populations.

## Introduction

A diversity of flow patterns in the ascending aorta (AA) has been documented in mainly patients with a bicuspid aortic valve [1–3]. Flow displacement (FD) with high velocity close to the vessel wall is a hallmark and has been proposed as a surrogate for increased wall shear stress that might serve as a quantitative parameter for risk-stratifying patients regarding development of AA aneurysm [4].

It is well known that patients with bicuspid aortic valve frequently develop AA aneurysms and the risk of aortic dissection or rupture is markedly increased compared with patients with tricuspid aortic valve [5]. Still, AA aneurysm is a frequent finding in patients with moderate and severe AR and, importantly, a majority of patients with dilated AA and AR have a tricuspid aortic valve [6]. Previous studies on the flow profile in the AA excluded [7–9] or had only a few [1, 3, 4, 10] patients with significant AR. From flow mechanics [11, 12] and previous studies [7, 13] it is conceivable that apart from valve phenotype we can expect that AA dilatation in itself and significant AR due to increased velocity are determinants of FD, but this has not been verified.

The high-resolution four-dimensional (4D) magnetic resonance imaging (MRI) acquisition needed for the most comprehensive description of flow is time-consuming and requires highly specialized expertise. The conventional method to measure aortic regurgitation (AR) from the amount of backward flow during diastole, is based on net through-plane phase contrast flow rate curves (PC-FRC). Such curves are derived from a cross-sectional plane typically in the proximal AA at the sino-tubular junction. Using the same cross sectional plane, the two-dimensional velocity profile across the vessel area for different phases of the cardiac cycle can be visualized (2D-PC). FD defined as the distance between the center of the lumen and the “center of velocity” normalized to the lumen radius can be described using conventional 2D-PC data [9]. Complex flow patterns leads to regions of simultaneous forward and backward flow across the vessel area [14, 15]. By decomposing the net PC-FRC, the forward and backward volume flow throughout the cardiac cycle can be visualized and quantified separately for systole and diastole [14]. In the present study, we selected patients with moderate to severe AR in order to investigate both forward and backward flow through the entire heart cycle. We hypothesized that the PC-FRC contains quantitative information about FD in both systole and diastole.

Thus, the aims of the study were threefold; (1) to study the systolic and diastolic flow profile in patients with moderate or severe AR, (2) to identify predictors of FD and (3) to investigate whether PC-FRC contain quantitative information related to FD.

## Materials and methods

### Study population

This prospective study comprised 40 patients with chronic AR and 22 controls. The patients were either investigated prior to valve surgery (n = 23) or as part of follow-up due to moderate (n = 14) or severe regurgitation (n = 3). Exclusion criteria were ≥moderate regurgitation in any other valve, presence of a cardiac shunt, any other form of significant cardiac disease and irregular heart rhythm. The controls were students or recruited from the hospital staff. They did not have any symptoms or history of cardiovascular disease.

The study was conducted according to the Declaration of Helsinki. The Regional Ethics Review Board gave ethical approval for the study protocol, and written informed consent was obtained from all participants.

### MRI examination

The MRI examination was performed on a 1.5 T scanner (Achieva, Philips Healthcare, Best, The Netherlands) using a five-channel phased-array cardiac coil. After standardized patient-specific planning, a series of cine-images was performed, first in the short-axis view covering the whole heart without gap (slice thickness 8 mm) from the atrioventricular ring to the apex, followed by cine-images in the standard long-axis projections including the left ventricular and right ventricular outflow tract view. All cine-images were acquired using balanced steady-state free precession sequences (repetition time 3.4 ms, echo time 1.7 ms, flip angle 60°) with retrospective ECG gating (30 phases per cardiac cycle) and parallel imaging (acceleration factor 2) during gentle expiratory breath-hold. Through-plane PC-MRI measurements (field of view = 320 × 260 mm, voxel size = 2.5 × 2.5 mm, flip angle = 12°, repetition time = 4.8 ms, echo time = 2.9 ms, receiver band width = 477.8 Hz/pixel, frames per heart cycle = 40, acceleration factor = 2, turbo field echo factor = 4, turbo field echo shots = 13, number of averages = 1) were obtained at the sino-tubular junction and in the pulmonary trunk just above the pulmonary valve.

### Analysis of the MRI data

Image analysis was performed using ViewForum (Philips Healthcare, Best, The Netherlands). Left ventricular volumes were obtained by manually tracing the endocardial contour in end-diastole in the successive short axis slices of the continuous short axis stack, propagated through all phases using a semi-automated tracing algorithm, followed by manual adjustment, if necessary. Conventional analysis of the PC measurements with net PC-FRC was performed using the integrated workstation of the MRI scanner (Easy Vision, software release 5.1.7.1, Philips Healthcare, Best, The Netherlands). The regurgitant volume was calculated as the difference between the left ventricular stroke volume and the pulmonary stroke volume. The regurgitant fraction was calculated as the regurgitant volume/left ventricular stroke volume ×100. The dimensions of the left ventricular outflow tract, sinus valsalva, sino-tubular junction and AA were assessed using a cine three chamber and the left ventricular outflow tract projection. On the basis of the AA diameter, the aorta was defined as normal (<40 mm) or dilated (≥40 mm) [16]. The pattern of AA dilatation was categorized in three phenotypes: no-dilatation phenotype, ascending phenotype (dilated AA with less dilated root), and root phenotype (dilated root with normal or less dilated AA) [17].

**Fig. 1:**
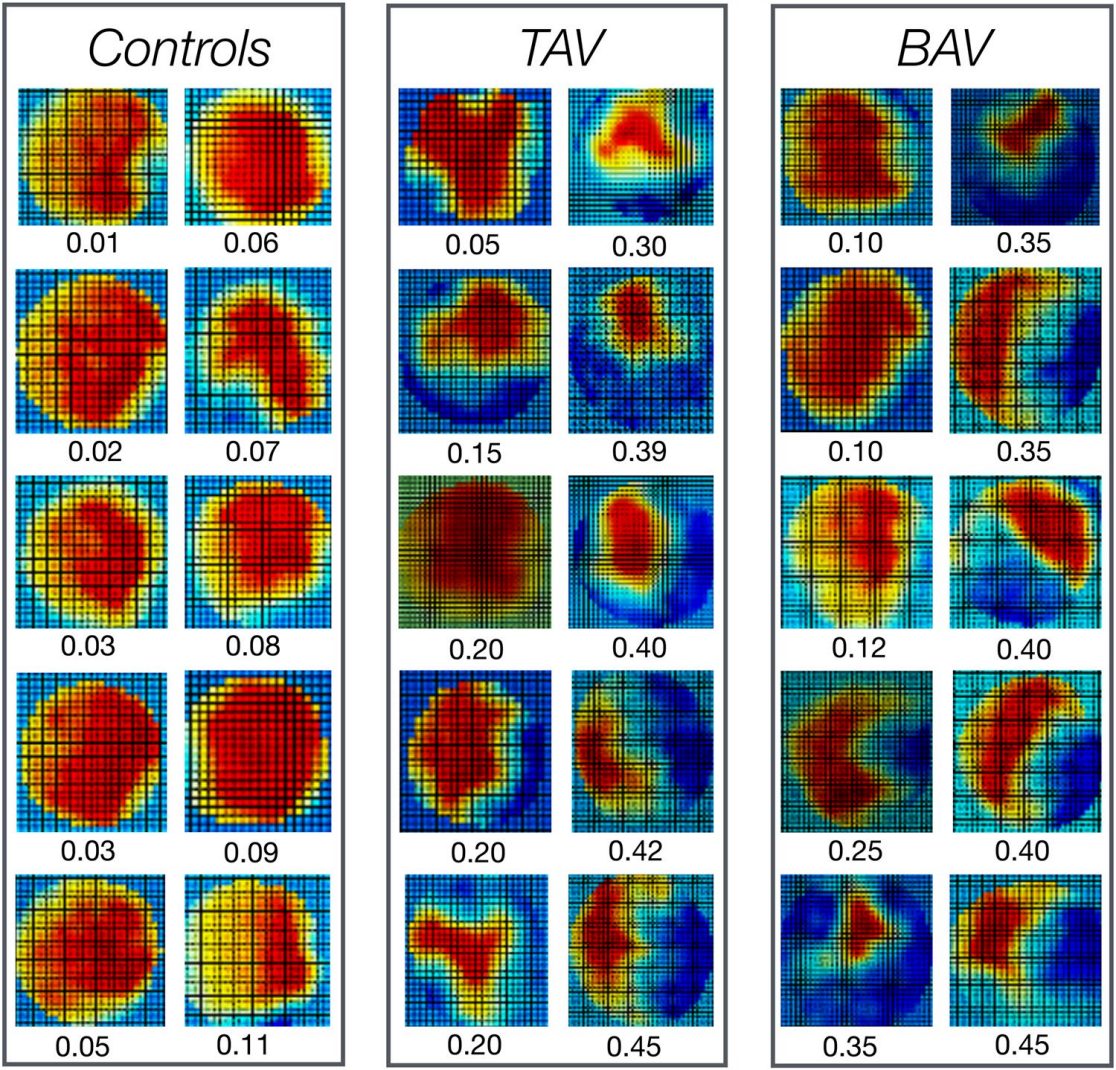
2D-PC cross-sectional peak systolic velocity patterns in 10 controls, 10 patients with tricuspid aortic valve (TAV) and ten patients with bicuspid aortic valve (BAV). Forward flow is *red* and backward flow is *blue*. (Color figure online)

The net PC-FRC, derived in the conventional analysis, was reanalyzed with a research software (Segment v1.9 R2046), [18] that enabled decomposition of the net flow into backward and forward flow. The systolic forward and backward flow volume was calculated from the systolic part of the forward and backward PC-FRC. The corresponding diastolic backward and forward flow volume was calculated similarly during diastole. Additionally, 2D velocity profiles describing the velocity distribution over the vessel area and cardiac cycle were plotted as color maps (Fig. 1). FD was quantified using a method previously described by Sigovan et al. [9] which defines FD as the distance between the center of the lumen and the ‘‘center of velocity’’ of the peak forward flow at systole and the peak negative velocity in diastole normalized to the lumen radius. The systolic and diastolic FD could due to limitations in spatial resolution, in most patients only be determined in intervals of 0.05.

### Statistical analysis

Continuous variables are expressed as the mean ± SD or median (range) when appropriate. The degree of linear relationship was assessed by the correlation coefficient (R). To compare multiple groups we used one-way ANOVA test when the distribution was normal or Kruskal–Wallis test when the distribution was not normal. In cases where the null-hypothesis was rejected (p value <0.05 considered statistically significant) we continued with a post-hoc analysis using the independent-sample *t* test or Mann–Whitney test when appropriate. Then comparisons between the three groups were performed and the p value adjusted to 0.016 using the Bonferroni correction. To study predictors of systolic and diastolic FD, simple and multiple regression analysis was used. To establish optimal cut-off values for detection of marked systolic and diastolic FD receiver operator characteristic curves (ROC) were used and the area under the curves (95% confidence interval) was determined. Diagnostic utility was described using sensitivity, specificity, positive and negative likelihood ratios [19]. Inter- and intra-observer variability was determined in 15 randomly selected investigations by two observers and assessed by the coefficient of variation (defined as the SD of the differences between observer measurements divided by the mean of the observer measurements). Statistical analysis was performed using IBM SPSS Statistics 23 (IBM Corporation, Somers, NY, USA).

## Results

The mean ± SD age of controls was 30 ± 7 years and 14 (64%) were females. All controls had tricuspid aortic valve, normal aortic dimensions and a normal aortic phenotype. Healthy volunteers did not have any systolic backward flow except in late systole.

The mean ± SD age of patients was 50 ± 15 years and 85% were male. Nineteen patients (47%) had a tricuspid aortic valve and 21 (53%) a bicuspid aortic valve. The AR mechanism was cusp prolapse in 28 patients (70%), annular dilatation in 5 patients (13%), cusp rupture or perforation in 4 patients (10%) and restricted cusp motion in 3 patients (7%).

### Flow profiles in the AA in controls and patients with AR

Table 1 compares clinical and CMR findings in patients with tricuspid aortic valve and bicuspid aortic valve. Patients with bicuspid aortic valve were younger than patients with tricuspid aortic valve and had higher left ventricular ejection fraction. The regurgitant volume did not differ significantly between the groups, but there was a tendency towards lower regurgitant fraction in patients with bicuspid aortic valve compared with tricuspid aortic valve. Patients with a bicuspid aortic valve had significantly larger sino-tubular junction compared with tricuspid aortic valve and there was a tendency towards larger AA. There was no significant difference in aortic phenotypes between AR patients with bicuspid aortic valve compared with tricuspid aortic valve. Patients with bicuspid aortic valve had comparable SFF and DBF but significantly larger systolic backward flow and diastolic forward flow compared with tricuspid aortic valve.

**Table 1:** Findings in patients with tricuspid aortic valve compared with patients with bicuspid aortic valve

Variables	Tricuspid aortic valve (n = 19)	Bicuspid aortic valve (n = 21)	*P* value
Age (years)	59 ± 15	43 ± 12	0.001
BSA (m^2^)	2.01 ± 0.28	2.05 ± 0.17	0.68
Systolic blood pressure (mmHg)	147 ± 27	130 ± 15	0.03
Diastolic blood pressure (mmHg)	65 ± 14	68 ± 13	0.5
Heart rate (bpm)	63 ± 9	61 ± 10	0.4
LV end-diastolic volume (ml)	332 ± 100	300 ± 94	0.30
LV end-systolic volume (ml)	151 ± 56	118 ± 44	0.046
Ejection fraction (%)	55 ± 7	61 ± 6	0.006
Regurgitant volume (ml)	95 (19–194)	79 (20–201)	0.23
Regurgitant fraction (%)	50 (21–73)	40 (15–73)	0.044
Left ventricular outflow tract (mm)	27 ± 3	32 ± 3	< 0.001
Sinus of valsalva (mm)	39 ± 5	42 ± 5	0.11
Sino-tubular junction (mm)	32 ± 6	36 ± 4	0.04
Ascending aorta (mm)	38 ± 8	43 ± 8	0.05
No-dilatation phenotype (no (%))	10 (53)	6 (29)	0.20
Ascending phenotype (no (%))	5 (28)	10 (48)	0.32
Root phenotype (no (%))	4 (22)	5 (23)	1.0
Peak aortic velocity (m/s)	2.0 ± 0.5	2.1 ± 0.6	0.67
Peak doppler gradient (mmHg)	17 (7–52)	19 (6–52)	0.69
Systolic forward flow (ml)	156 (91–243)	181 (124–355)	0.44
Systolic backward flow (ml)	9 (0–41)	35 (1–71)	0.004
Diastolic backward flow (ml)	81 (24–141)	76 (30–208)	0.65
Diastolic forward flow (ml)	6.5 (1–28)	21 (1–53)	0.007

Mean ± SD or median (range) when appropriate

*BSA* body surface area, *LV* left ventricular

**Fig. 2:**
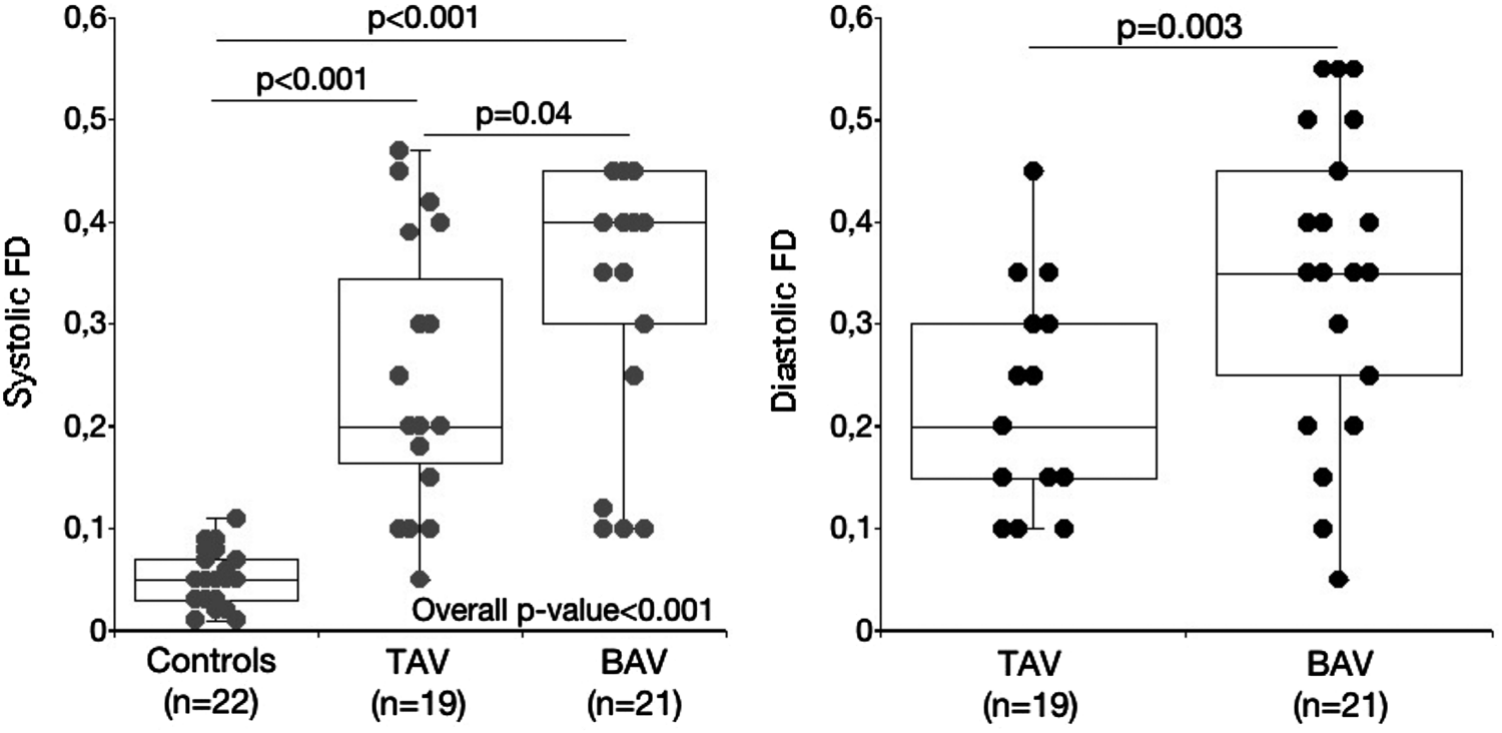
Box plots showing the systolic FD in controls, patients with tricuspid aortic valve (TAV) and bicuspid aortic valve (BAV) (*left*) and the diastolic FD in patients with TAV and BAV

Figure 1 shows the 2D velocity profiles at peak systolic flow in 10 controls, ten patients with tricuspid aortic valve and ten with bicuspid aortic valve. The value of the systolic FD is shown for each individual. Note that the actual size of the aorta is not presented, all vessels appear to have the same size. The typical 2D velocity profile in a normal control is a centrally located systolic outflow jet with uniform velocity distribution and only forward flow during systole. In contrast, patients with AR may have large areas of the vessel with systolic backward flow. The shape of the systolic forward flow velocity contour varies markedly both in patients with tricuspid aortic valve and bicuspid aortic valve. Patients with similar degree of marked systolic FD have generally small areas of systolic forward flow but the degree of crescent-shaped attachment to the vessel wall differs considerably. The systolic FD tended to be (p = 0.04) more pronounced in patients with bicuspid aortic valve compared with tricuspid aortic valve (Fig. 2). The diastolic FD was more pronounced in patients with bicuspid aortic valve compared with tricuspid aortic valve (Fig. 2).

**Fig. 3:**
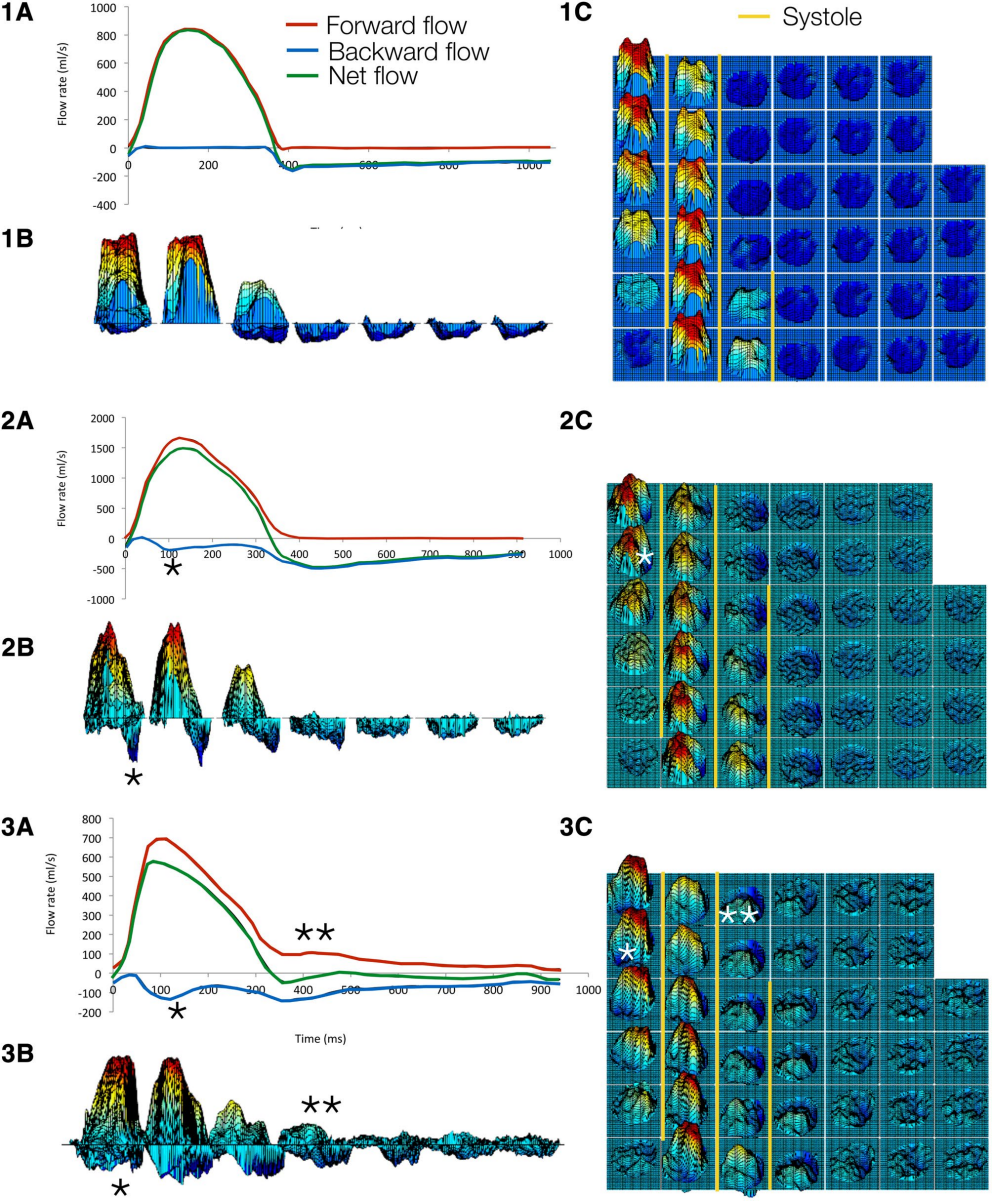
PC-FRC **a** and 2D PC flow profiles (**b**,** c**) from three patients describing the three typical flow patterns observed. The *upper panel* shows a patient (Group 1) with tricuspid aortic valve and severe AR due to rupture of a cusp. The AA diameter was 27 mm. There were no systolic backward flow and no diastolic forward flow. The *middle panel* shows a patient (Group 2) with bicuspid aortic valve and severe AR due to prolapse of a cusp. The AA diameter was 47 mm. Note that there was systolic backward flow (*) but no diastolic forward flow. The *lower panel* shows a patient (Group 3) with bicuspid aortic valve and moderate AR due to prolapse of a cusp. The bicuspid aortic valve phenotype was fusion of the right and non-coronary cusp. The AA diameter was 47 mm. Note that there was systolic backward flow and diastolic forward flow (**)

Figure 3 shows three typical PC-FRCs where the net flow was decomposed into forward- and backward flow separately during the cardiac cycle. The corresponding flow patterns are also shown in terms of 2D-PC velocity profiles. Areas of forward flow velocity are depicted with red color and areas with backward flow with blue color. Twelve patients (30%, Group 1) had PC-FRC with only forward flow in systole and backward flow in diastole. Ten patients (25%, Group 2) had backward flow in systole beginning earlier than the time point of the peak systolic flow. A region of eccentric systolic forward flow and a region of systolic backward flow characterized the 2D velocity profiles. The remaining 18 patients (45%, Group 3) had systolic backward flow as Group 2, but also PC-FRC with diastolic forward flow. The diastolic backward flow and diastolic forward flow were typically present at separate regions of the vessel lumen (Fig. 3, patient 3). Patients in Group 1 had more severe regurgitation and no dilatation of the AA compared with Group 2 and Group 3 (Table 2). Patients in Group 3 and Group 2 had comparable degree of regurgitation but Group 3 had more often bicuspid aortic valve, significantly larger AA and more pronounced FD both in systole and diastole.

**Table 2:** Comparison between three different PC-FRC patterns

Variables	Group 1 (*n* = 12)	Group 2 (*n* = 10)	Group 3 (*n* = 18)	Overall *P*	Post hoc analysis		
					Group 1 vs Group 2	Group 1 vs Group 3	Group 2 vs Group 3
Bicuspid aortic valve (n (%))	4 (30)	3 (30)	14 (70)	*	–	–	–
AA dimension (mm)	34 ± 4	37 ± 6	47 ± 6	<0.001	0.11	<0.001	< 0.001
Regurgitant volume (ml)	120 (39–185)	68 (32–201)	62 (19–194)	0.005	0.006	0.002	0.55
Regurgitant fraction (%)	59 (29–69)	40 (26–57)	37 (15–73)	0.009	0.006	0.007	0.58
Systolic backward flow (ml)	5 (0.3–13)	17 (5–39)	38 (20–71)	<0.001	<0.001	<0.001	0.001
Diastolic forward flow (ml)	2 (1–7)	9 (4–11)	26 (11–53)	<0.001	<0.001	<0.001	< 0.001
Systolic flow displacement	0.10 (0.05–0.25)	0.28 (0.15–0.40)	0.41 (0.30–0.47)	<0.001	0.001	<0.001	0.001
Diastolic flow displacement	0.15 (0.05–0.20)	0.25 (0.10–0.40)	0.38 (0.30–0.55)	<0.001	<0.001	<0.001	< 0.001

Mean ± SD or median (range) when appropriate

*AA* ascending aorta

*To small number of observations to allow statistical calculations

### Predictors of FD

The AA diameter independently predicted the systolic FD (Table 3), while the AA diameter and regurgitant volume independently predicted the diastolic FD. Valve phenotype was not an independent predictor of systolic or diastolic FD.

**Table 3:** Factors affecting systolic and diastolic FD

*Simple regression*	Systolic FD				Diastolic FD			
	*R*			*P*	*R*			*P*
Ascending aorta diameter (mm)	0.70			<0.001	0.61			<0.001
Regurgitant volume (ml)	0.28			0.08	0.48			0.002
Valve phenotype (bicuspid = 1)	0.35			0.03	0.45			0.003

*Multiple regresion*		*B*	Std error	*P*		*B*	Std error	*P*
Ascending aorta diameter (mm)		0.011	0.002	< 0.001		0.010	0.002	< 0.001
Regurgitant volume (ml)						−0.001	0.0003	< 0.001
Valve phenotype (bicuspid = 1)		0.038	0.32	0.25		0.059	0.03	0.058
Regression equation	0.72				0.80			

*R *correlation coefficient,* B * slope of the regression line

### Relationship between PC-FRC and 2D-PC flow profile data

**Fig. 4:**
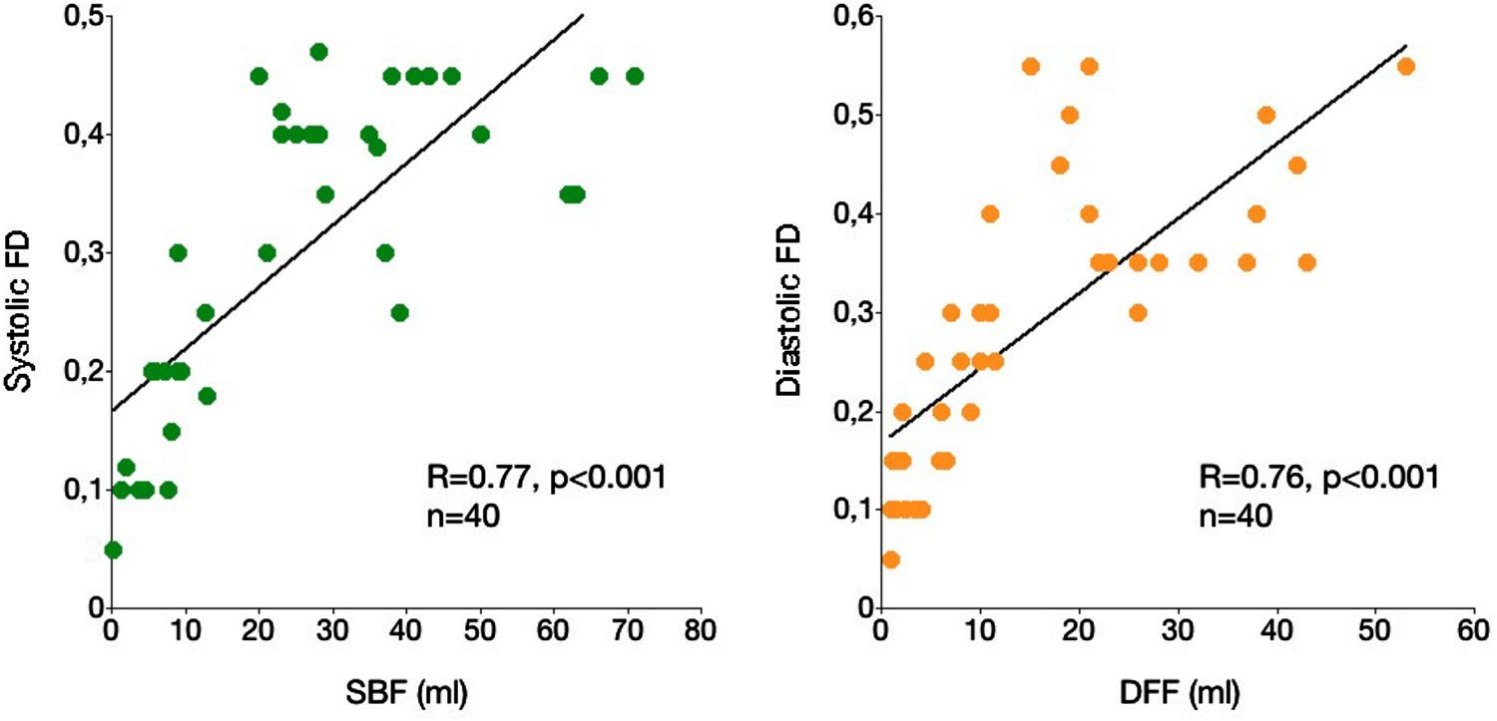
Scatterplots showing the relationships between systolic backward flow (SBF) and systolic FD (*left*) and diastolic forward flow (DFF) and negative diastolic FD (*right*)

The linear relationship between systolic backward flow estimated from PC-FRC and systolic FD estimated from 2D-PC was strong (Fig. 4). The corresponding relationship between diastolic forward flow and negative diastolic FD was strong (Fig. 4).

**Fig. 5:**
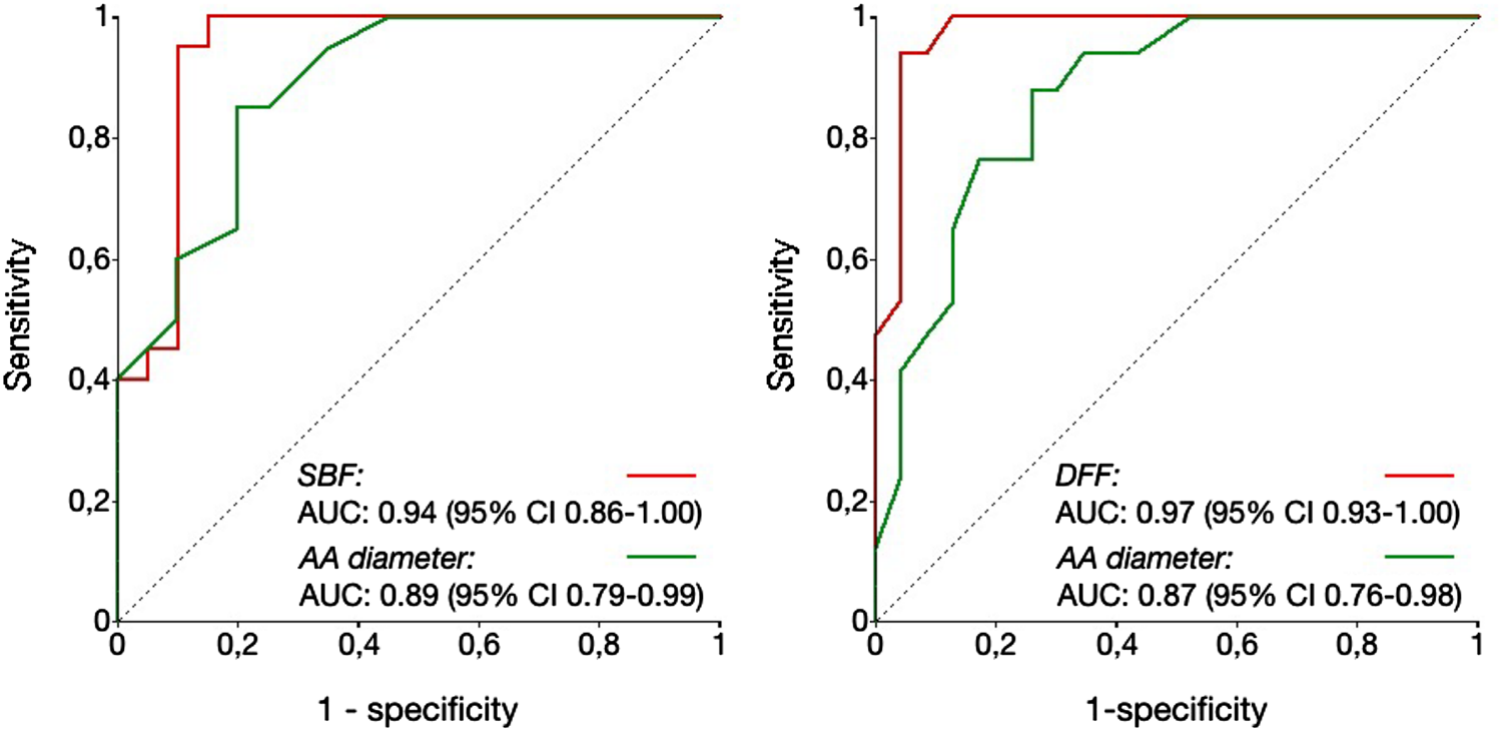
ROC *curves* for the detection of marked systolic (*left*) or diastolic (*right*) FD (≥0.35)

Figure 5 shows ROC curves for systolic backward flow and AA diameter for the detection of marked systolic FD (≥0.35) and ROC curves for diastolic forward flow and AA diameter for the detection of marked diastolic FD (≥0.35), respectively. The areas under the curve were larger for PC-FRC-based volume flow parameters compared with AA diameter. Table 4 shows the diagnostic performance of different cut-off values. With systolic backward flow volume above the cut-off value the likelihood of marked systolic FD increased sixfold. With diastolic forward flow volume above the cut-off value the likelihood of marked diastolic FD increased 10.8 fold.

**Table 4:** Diagnostic performance regarding the detection of marked systolic and diastolic FD (≥0.35)

Variable	Cut-off	Sensitivity (95% CI)	Specificity (95% CI)	PLR (95% CI)	NLR (95% CI)
Systolic FD					
AA diameter (mm)	≥40	80 (58–92)	80 (58–92)	4.0 (1.6–9.9)	0.25 (0.1–0.6)
SBF (ml)	≥21	95 (76–99)	84 (62–94)	6.0 (2.1–17.1)	0.06 (0.01–0.4)
Diastolic FD					
AA diameter (mm)	≥40	82 (59–94)	74 (54–87)	3.2 (1.5–6.5)	0.24 (0.08–0.69)
DFF (ml)	≥12	94 (73–99)	91 (73–98)	10.8 (2.9–41)	0.06 (0.01–0.43)

*CI* confidence interval, *AA* ascending aorta, *PLR* positive likelihood ratio, *NLR* negative likelihood ratio, *SBF* systolic backward flow, *DFF* diastolic forward flow

### Inter- and intra observer variability

The intra-observer variability for regurgitant volume and regurgitant fraction was 0.2 and 0.5% respectively. The corresponding inter-observer variability was 0.3 and 0.6% respectively.

## Discussion

In the present study, we found that conventional PC-FRC provide continuous variables that are related to the complexity of flow in patients with AR and that PC-FRC data can be used to detect marked FD in the AA. Complex flow patterns are common findings in patients with bicuspid aortic valve but they are also present in patients with tricuspid aortic valve when the AA is dilated.

Dissection of the AA is a medical condition with high acute phase mortality and high operative mortality despite surgical improvements [20]. There are several studies indicating that the risk of aortic dissection is relatively low when AA diameter <50 mm but with a marked increase in risk when AA diameter >60 mm [5, 21, 22]. However, it has been estimated that 60% of non-Marfan patients with aortic dissection and tricuspid aortic valve did not have a dilated AA prior to the dissection and only 3% fulfilled criteria (>55 mm) for elective AA replacement [23]. Thus, there is a knowledge gap regarding predictors of AA dissection, especially in patients with AA < 55 mm [22]. The most common risk factor associated with AA dissection is hypertension, however, considering the high prevalence in the general population, hypertension in itself is not useful in identifying individuals at risk [20]. Furthermore, bio- or genetic markers related to development of thoracic aortic disease are still not available [24]. More recently, in search for reliable risk predictors beyond AA dimension and aortic valve phenotype, the development of 4D MRI techniques has enabled visualization and quantification of flow abnormalities and FD with regional increased wall stress, which has been suggested as a physiological mechanism explaining the observed AA phenotype in patients with bicuspid aortic valve [1–3]. However, the advanced 4D MRI technique is time-consuming and complex and therefore not feasible to use in large patient populations. Therefore, our findings that conventional PC-FRC contains information about the complexity of flow profiles and that patients with marked FD can be identified is interesting in this context. Today, the option to decompose a net PC-FRC is available in some but not all MRI-scanners. However, the software needed to perform the analysis is a freely available research tool [18]. Our findings suggest that conventional PC-FRC has the potential to be a research and screening tool but further studies in larger populations are warranted to elucidate whether FD is related to AA growth and/or aortic dissection or rupture.

Unlike previous studies investigating flow pattern and wall stress in AA, the present study included patients with isolated AR without valve calcification or important stenosis. The valve opening in the patients with tricuspid aortic valve is symmetrical and, therefore, it was an unexpected finding that patients with tricuspid aortic valve also may have complex flow patterns with FD and systolic backward flow at the sino-tubular junction. In the present study, we found that AA dimension was the strongest predictor of systolic FD but that bicuspid aortic valve was not an independent predictor. These findings are seemingly in contrast to other studies that have highlighted the differences between bicuspid aortic valve and tricuspid aortic valve [1, 3, 8, 25]. Mahadevia et al. used aorta size-matched controls as a reference group and concluded that they, like healthy controls, did not have abnormal FD in comparison with patients with bicuspid aortic valve [3]. However, it is notable that in their study FD was significantly higher in aorta size-matched controls with tricuspid aortic valve in comparison to healthy controls indicating some degree of abnormality although less than patients with bicuspid aortic valve [3]. Although the present study was not designed to elucidate physiologic mechanisms behind the observed eccentricity of flow close to the aortic valve, there are several contributing factors to be considered. These include the flow profile in the left ventricular outflow tract, the position of the valve orifice in the vessel cross-sectional area, the opening of the cusps, and the size and the shape of the aorta. The velocity profile is known to be eccentric in the left ventricular outflow tract with highest velocity in the anteroseptal part of the cross-sectional area in normal subjects [26]. Therefore, a slight asymmetry present in the flow profile in the aortic root is also present in patients with tricuspid aortic valve. This asymmetry will be more pronounced in patients with bicuspid aortic valve because the orifice is often anatomically eccentric and there might be restrictions in cusp motion that directs the flow toward the aortic wall. Basing on fluid mechanic theory the importance of the ratio between the valve area or area of forward flow and the AA area is recognized and dilatation favors turbulence [12]. The velocity in the forward systolic jet is higher than in the surrounding part with turbulent or backward flow. According to the Bernoulli’s principle the pressure will be lower in the high-velocity compared with a low-velocity region and, therefore, the arising pressure gradient will push the eccentric outflow jet towards the aortic wall. Hence, substantial FD can be expected regardless of valve phenotype in patients with dilated AA.

The 2D-PC velocity profiles in patients with dilated AA showed backward flow in a large part of the trans-sectional area of the vessel during the systolic period. In patients with dilated AA and moderate to severe AR there was a large eccentric area of forward flow during diastole. This suggests that there are large flow vortices established in the dilated AA and not simply chaotic turbulent flow. To what extent this complex flow in both systole and diastole influences the PC assessment of stroke volume, regurgitant volume and regurgitant fraction is an important issue. The conventional PC method for grading of AR severity utilizes the through-plane velocity vector for calculation of the flow volume. With complex flow it is conceivable that the perpendicular velocity vector underestimates the true velocity and thereby the true volume flow. One study by Muzzarelli et al. support this conclusion and showed that the stroke volume based on PC measurements was underestimated in the AA in patients with bicuspid aortic valve and dilated AA compared with measurements in normal controls with tricuspid aortic valve [27]. The study did not address the problem regarding assessment of AR severity. The PC method with the through-plane level at the sino-tubular junction or proximal AA is the most commonly used [28] and therefore further studies are warranted to elucidate how complex flow can influence the grading of AR severity.

The limitations of the present study are related to (1) the small number of patients, (2) that only patients with moderate or severe AR were studied, (3) that we do not have any 4D MRI data for comparison and (4) that we did not perform a true test of reproducibility. We performed ROC analysis to determine the ability of PC-FRC data to detect marked FD. With a larger study population we could have established cut-off values in a derivation group and evaluated them in a test group. Whether the findings regarding the relation between PC-FRC and FD by 2D-PC in systole in patients with AR can be extrapolated to patients without AR is an important issue. The peak velocity in the AA by echocardiography (Table 1) was only slightly elevated in AR patients compared with what is observed in normal individuals and, therefore, a more pronounced Bernouilli effect in AR patients due to high velocities is not likely. Still, a control group with dilated AA, tricuspid aortic valve and without AR would have strengthened our results. In the present study we did not have 4D MRI data for comparison. This is not a limitation regarding the assessment of FD because the method used also in 4D MRI studies, is based on 2D-PC data [9]. Still, with 4D MRI data we could have investigated the relation between FD, valve phenotype and wall shear stress. We did perform an inter- and intra-observer variability test that showed extremely good reproducibility. These data should, however, be interpreted with caution because they are an expected finding considering the semiautomatic procedure. A test–retest design that implies taking the patient out of and then in again to perform a new acquisition is far more informative but with MRI this is time consuming and difficult to conduct.

## Conclusions

We have shown that conventional PC-FRC can be used to quantify and detect individuals with marked FD, which has been suggested as a risk marker of AA expansion in previous studies. Valve phenotype is not an independent predictor of systolic or diastolic flow displacement. The possible diagnostic and prognostic importance of FD is yet to be validated in larger patient populations. Our findings suggest that PC-FRC can be an alternative to the highly technically demanding 4D MRI method.
